# Cross‐Cultural Adaptation and Cross‐Validation of the Italian Version of the EPICC Spiritual Care Competency Self‐Assessment Tool for Clinical Nurses

**DOI:** 10.1111/jocn.17738

**Published:** 2025-03-18

**Authors:** Michela Piredda, Alessio Lo Cascio, Maddalena De Maria, Roberto Latina, Anna Marchetti, Anna De Benedictis, Giorgia Petrucci, Linda Ross, Wilfred McSherry, Maria Grazia De Marinis

**Affiliations:** ^1^ Department of Medicine and Surgery, Research Unit Nursing Science Campus Bio‐Medico di Roma University Rome Italy; ^2^ Department of Biomedicine and Prevention University of Rome Tor Vergata Rome Italy; ^3^ Department of Life Science, Health, and Health Professions Link Campus University Rome Italy; ^4^ Department of Health Promotion, Mother and Child Care, Internal Medicine and Medical Specialties University of Palermo Palermo Italy; ^5^ Department of Medicine and Surgery Fondazione Policlinico Universitario Campus Bio‐Medico Rome Italy; ^6^ School of Care Sciences, Faculty of Life Sciences & Education University of South Wales Wales UK; ^7^ Department of Nursing, School of Health, Education, Policing and Sciences University of Staffordshire Staffordshire UK

**Keywords:** EPICC tool‐it, nursing competencies, psychometric testing, spiritual care competencies

## Abstract

**Aim:**

To cross‐culturally adapt and psychometrically test the Italian version of the EPICC Spiritual Care Competency Self‐Assessment Tool for clinical nurses (EPICC Tool‐It).

**Design:**

Multicentre, cross‐sectional validation study.

**Methods:**

The 28‐item EPICC Tool was translated into Italian and culturally adapted following a rigorous methodology. A nationwide survey was conducted. Psychometric evaluation included content validity, structural validity (exploratory and confirmatory factor analyses), construct validity (known group analysis) and reliability using Cronbach's alpha, McDonald's omega and factor score determinacy.

**Results:**

The sample included 725 clinical nurses (76% female, 80% hospital‐based), on average 38.7 years old (SD 11.33), with 14.6 years (SD 11.03) of experience. Confirmatory factor analysis supported a four‐factor model (*Knowledge of spirituality*, *Attitudes towards spirituality and spiritual care*, *Knowledge of spiritual care* and *Skills in spiritual care*), with a second‐order factor for the EPICC Tool‐It. Construct validity was supported through known group analysis, showing score variation based on nurses' experience, education and religiosity. Internal consistency was excellent across all factors and the overall scale.

**Conclusion:**

A valid, multidimensional instrument is provided to assess spiritual care competencies in Italian‐speaking nurses. The EPICC Tool‐It is suitable for research and practice, facilitating evaluation of self‐perceived competencies and educational effectiveness.

**Implications for the Profession and/or Patient Care:**

The use of the EPICC Tool‐It by nursing managers, educators and clinicians is recommended in both clinical and research settings to support education on spiritual care competencies.

**Impact:**

The EPICC Tool‐It sets reliable measurement standards for spiritual care competencies, enhancing holistic care and comprehensive understanding of competencies globally.

**Reporting:**

This study adheres to the Strengthening the Reporting of Observational studies in Epidemiology (STROBE) guidelines.

**Patient or Public Contribution:**

Patients, service users, caregivers, or the public were not involved in the study. However, nurses as target users of the tool participated in the cultural adaptation and validation process.

**Summary**

- What does this paper contribute to the wider global clinical community?
  ○ It provides a valid and reliable tool for assessing nurses' self‐perceived spiritual care competencies in the Italian context, addressing a critical gap.
  ○ The study outlines a cross‐cultural adaptation and validation process, which can be replicated to validate the EPICC Tool in other cultures and countries.
  ○ It underscores the importance of assessing and enhancing spiritual care competencies, an indispensable element of holistic, patient‐centred nursing care.

## 1 Introduction

Spirituality is a fundamental aspect of individual identity and an indispensable component of integral patient care. It is a broad and unique concept, encompassing a variety of beliefs, values and practices that may or may not be associated with or inspired by faith traditions. Beliefs, values and practices that may or may not be associated with faith traditions. Existential concerns fall under the expansive umbrella of the spiritual domain (de Brito Sena et al. 2021; Ross et al. 2022; Ross and Austin 2015).

Improving spirituality and spiritual well‐being of patients is associated with a range of positive outcomes, including enhanced tolerance for the emotional and physical demands of illness, decreased pain, stress and negative emotions, and a reduced risk of depression and suicide among patients (Balboni et al. 2013; Heidari et al. 2021; Ross 1997). Additionally, patients receiving adequate spiritual care tend to report higher satisfaction with hospital care and treatments (Chen et al. 2018). An increasing inclination towards spirituality among patients is observed, who anticipate healthcare providers to address their spiritual needs (Best et al. 2024). While many healthcare providers recognise the needs and expectations of patients, spiritual care is not commonly perceived as an integral component of healthcare. In fact, only a small percentage of patients, ranging from 6% to 28%, receive spiritual care from their healthcare teams (Astrow et al. 2007; Selman et al. 2018). Unfulfilled spiritual needs impact patient well‐being, with adverse outcomes including reduced quality of life, increased risk of depression and diminished perception of spiritual peace (Connolly and Timmins 2021), dissatisfaction with care, reduced utilisation of hospice services, increased use of aggressive treatments and higher costs (Gijsberts et al. 2019; Salari et al. 2023). This incongruity between patient wishes and actual practice contradicts international policy guidance (Attard et al. 2019a; O'Brien et al. 2019).

The significance of spiritual, religious and cultural aspects on individuals' well‐being suggests that caring professions should receive education in this regard (de Brito Sena et al. 2021). Among the caring professions, nurses have spiritual care explicitly incorporated into the professional Codes of Ethics (International Council of Nurses 2021). Although clinical nurses consider spiritual care as part of their everyday practice, evidence indicates that they feel unprepared for it and request more education (Attard et al. 2019b; Egan et al. 2017; McSherry 2010; Rykkje et al. 2022; Ross et al. 2018).

The primary cause of this shortfall lies in the lack or inadequacy of nurses' abilities to deliver spiritual assistance. It is therefore crucial to know and nurture nurses' competence in spiritual care as a prerequisite for providing such care, for which a variety of training programmes have been devised and implemented (Amiri et al. 2021; Giske et al. 2023; Musa et al. 2023).

## 2 Background

Spirituality plays a pivotal role in integral patient‐centred care (Gijsberts et al. 2019; Puchalski et al. 2014; Southard 2020). Nevertheless, hospitalised individuals report that nurses seldom address spiritual concerns, and spiritual care remains infrequent irrespective of their diagnosis (Balboni et al. 2013; Caldeira et al. 2017; Selman et al. 2018). Patients might not always tell nurses and other healthcare professionals about their spiritual needs, so those needs might be overlooked. Moreover, global reports indicate that nurses routinely disregard the spiritual aspect, even in the presence of cues indicative of deep inner concerns (Mthembu et al. 2016). Dealing with spirituality in healthcare is tricky because it is hard to measure and it is difficult to learn how to care for people's spiritual needs properly (Cone and Giske 2017; Rykkje et al. 2022; Weathers et al. 2016). Moreover, the provision of spiritual care encounters several obstacles (Cone and Giske 2021; Kuven and Giske 2019; Neathery et al. 2020). In fact, healthcare professionals, including nurses, often express uncertainty regarding the nature of spirituality, report having received limited training in spiritual care (Cone 2019; Giske and Cone 2012; Ødbehr et al. 2015), and feel ill‐prepared to provide spiritual care (McSherry and Jamieson 2013; Minton et al. 2018).

Education and training on spirituality can enhance spiritual competencies, foster individual spiritual growth and amplify the ability to provide patient‐centred whole‐person care (Giske and Cone 2012; McSherry et al. 2020; Ross et al. 2018; Rykkje et al. 2022; Wu et al. 2016).

Recently a team of scholars from Europe, Africa and the United States developed a self‐assessment measure of spiritual care competence named ‘Enhancing Nurses' and Midwives' Competence in Providing Spiritual Care through Innovative Education and Compassionate Care’ (EPICC Tool) (McSherry et al. 2020; Ross et al. 2014). The EPICC Tool builds on and advances the seminal work of van Leeuwen et al. (2021) and Attard et al. (2019a) since the EPICC tool is a synthesis of their work, which formed the basis of developing the EPICC Spiritual Care Education Standard referred to here as the EPICC Standard (van Leeuwen et al. 2021). Therefore, the 28‐item EPICC Spiritual Care Competency Self‐Assessment Tool that was constructed from the EPICC Spiritual Care Education Standard is the most up to date tool that assess student nurses' and nurses' perceived competence in the delivery of spiritual care (Giske et al. 2023). The EPICC Tool was psychometrically tested for validity and reliability (Giske et al. 2023). However, the use of validated instruments in different languages and cultural contexts, despite being desirable for comparison, cannot be assumed only by their translations (De Maria et al. 2021). The use of the same instrument in different contexts requires rigorous justification.

## 3 Aim

The aim of this study was to conduct an Italian cultural adaptation of the EPICC Tool for self‐assessment of spiritual care competence (referred to as EPICC Tool‐It) and to test its psychometric properties (validity and reliability) with nurses.

## 4 Methods

### 4.1 Design

The design adopted was a multicentre cross‐sectional study. The Strengthening the Reporting of OBservational studies in Epidemiology (STROBE)—Checklist of items that should be included in reports of cross‐sectional studies (von Elm et al. 2014) was followed for conducting and reporting this study (Supporting Information 1).

### 4.2 Measurement

The 28‐item EPICC Tool (Giske et al. 2023) is a self‐assessment tool investigating four main competencies: (1) INTRApersonal Spirituality—addressing self‐understanding in the spiritual domain, (2) INTERpersonal Spirituality—focusing on relationships and connections between oneself and others, (3) Spiritual Care Assessment and Planning—adapting the first three steps of the Nursing Process where nurses assess and identify the problem and create an action plan and (4) Spiritual Care Intervention and Evaluation—where nurses implement the plan, evaluate it, and document what is important to convey to other members of the healthcare team. Each main competence includes items able to assess knowledge, skills, and attitudes in spiritual care. The psychometric properties of the EPICC Tool were tested in an international mixed‐methods study with 323 nursing/midwifery students (Giske et al. 2023) by conducting exploratory factor analysis and confirmatory factor analysis. The confirmatory factor analysis positing the four‐factor model mirroring the main competences that guided the instrument development yielded poor to acceptable fit indices and several items loading < 0.30. The Cronbach's alpha coefficient for the individual factors was between 0.7 and 0.8 and for the whole tool was 0.91. The tool was revised considering the open comments from students and statistical results. Helpful information such as definitions of spirituality and spiritual care, were added, and some items were reworded. The 4‐point Likert scale was changed to a 5‐point Likert scale from 1 = completely disagree to 5 = completely agree.

Additionally, a demographic questionnaire was administered to gather information on participants' socio‐demographic and professional characteristics, including gender, age, educational background, work experience, clinical context, geographic location and religious attendance.

### 4.3 Instrument Translation and Cultural Adaptation

The original EPICC Tool (Giske et al. 2023) was translated and culturally adapted following the steps recommended by the EPICC Steering Group based on international guidelines (Beaton et al. 2000; Martins et al. 2015). In step 1‐Forward Translation, the EPICC Tool was independently translated from English into Italian by two translators familiar with both languages and with the objectives of the instrument. In step 2‐Synthesis, the project leader and translators examined the initial translations (T1, T2) against the original instrument and produced a common version (T 1–2). Step 3‐Back Translation involved two professional translators, both unfamiliar with the tool and the objectives of the study, separately translating the T 1–2 version back into English. In step 4‐Review, the two back translations were sent to two members of the EPICC Steering group as instrument developers (Professors Linda Ross and Wilfred McSherry) who reported on which translation seemed more accurate and whether the item content retained conceptual equivalence with the original EPICC Tool. Their reports, together with all the translations, were examined by a panel of 10 experts from three Italian Universities including three Nursing Faculty, a methodologist, a Professor of Moral Philosophy, two nurses with PhD and three nurses with Master's degrees. The panel assessed semantic and conceptual equivalence between the source and the target instrument and produced a prefinal Italian version.

### 4.4 Study Setting and Sampling

Nurses were recruited using a combination of convenience and snowball sampling. Initially, convenience sampling was carried out through the personal networks of the researchers across multiple Italian regions. Snowball sampling was then employed to expand participation, with respondents encouraged to share the survey link with eligible colleagues, thereby ensuring broader geographic representation. The sample inclusion criteria were clinical nurses actively engaged in patient care across different settings (hospital or community) and clinical areas. Nurses not directly involved in patient care were excluded.

A sample of 280 subjects was judged to be sufficient based on the standard minimum requirement of at least 10 subjects per item to perform a confirmatory factor analysis (Kyriazos 2018). However, a larger sample was recruited to cover a nationally widespread distribution and enable between‐groups testing (MacCallum et al. 1999).

### 4.5 Data Collection

The link to the online questionnaire was distributed via email to potential participants between August 2022 and May 2023. Participants were provided with detailed information about the study's objectives and procedures and were invited to complete the questionnaire and share the link with their colleagues. The questionnaire was completed and returned online. To minimise the risk of missing or incorrect data, participants were required to select answers from predefined options, and all items in the questionnaire were mandatory. The form did not collect participants' email addresses or names, ensuring that individual participants could not be identified by the researchers.

### 4.6 Statistical Data Analysis

Descriptive analyses for the socio‐demographic variables of the sample and for the items of the EPICC Tool were conducted with SPSS v.28.00 (IBM Corp. Armonk, NY), while factor analyses were conducted with MPlus v.8.1 (Muthén and Muthén, Los Angeles, CA, USA). Skewness and kurtosis indices were calculated to evaluate the normality of item distribution (Muthén and Kaplan 1985). Structural validity was first tested through confirmatory factor analysis (CFA), positing the model of the original instrument for the entire sample. It was hypothesised that the CFA would confirm the four‐factor structural model, with factor loadings and inter‐item correlations reflecting such domains.

Given that the theoretical model did not adequately fit the data, we opted for an exploratory approach to address the issue of the number of latent dimensions underlying the items. Initially, parallel analysis was performed on the entire sample to determine the number of plausible factors for extraction. Once the optimal number of factors was defined, exploratory factorial analysis (EFA) was conducted. To evaluate the generalisability of the results, a cross‐validation method was used. The total sample was randomly split into two equivalent subsamples. The first subsample was used for the validation phase with EFA, while the second subsample served as the calibration set to replicate the factor structure with CFA (Kline 2023). With the EFA, we hypothesised that the covariances or correlations among a set of observed variables can be explained by a smaller number of unobservable latent factors. These latent factors are hypothesised to represent underlying constructs that influence the observed variables. Then, it was hypothesised that the CFA in a sample of 384 participants would confirm a four‐factor structural model, with factor loadings and inter‐item correlations reflecting the domains identified in the EFA. Furthermore, goodness‐of‐fit indices would demonstrate acceptable model fit in a sample of 346 participants (e.g., RMSEA < 0.08, CFI > 0.90). After establishing a replicable factor structure, we hypothesised the presence of a second‐order factor due to the observed correlations between the factors. Data factoriability was preliminarily assessed with the Kaiser‐Meyer‐Olkin (KMO) measure of sampling adequacy (values > 0.7 mean adequate factoriability) and the Bartlett's test of sphericity (must be significant).

Since the items were not normally distributed, consistent with Muthén and Muthén (2017), we used a robust maximum likelihood estimator (ML‐R estimator). According to recommendations by Hoyle (1995) and to a multifaceted approach to model fit testing (Bentler and Hu 1998), we used several fit indices including: the root mean square error of approximation (RMSEA), the Comparative Fit Index (CFI), the Tucker and Lewis Index (TLI) and the standardised root mean square residual (SRMR). Values of RMSEA ≤ 0.06; RMSEA with 90% confidence intervals ≤ 0.05 to ≤ 0.08; RMSEA test of close‐fit examining the probability that the approximation error is low *p* > 0.05; CFI/TLI > 0.95; and SRMR ≤ 0.08 indicate a good fit (Browne and Cudeck 1992; Hu and Bentler 1999). The *χ* ^2^ statistics were also computed and interpreted together with the above indices. The factor loadings >|0.30| were deemed adequate (Tabachnick and Fidell 2019).

Construct validity of the EPICC Tool‐It was evaluated through assessing score distribution among known groups by posing the following hypotheses in accordance with previous literature (Hsieh et al. 2020; Kang et al. 2021): (a) scores will be higher in more experienced compared with less experienced nurses; (b) scores will be higher in nurses with postgraduate education (i.e., master, PhD) compared with nurses with bachelor's; (c) scores will be different in nurses working in different clinical areas; (d) scores will be higher in participants reporting to practice a religion compared with people who did not report it. To this end, the respondent's work experience was classified as high or low based on the median (12 years). To assess such differences between scores, one‐way ANOVA or chi‐square test was performed according to the variables involved.

Reliability, in terms of internal consistency, was assessed. Since Cronbach's Alpha (*α*) is the most commonly reported coefficient, we calculated it for completeness; however, as the scale is multidimensional, a more appropriate coefficient such as the McDonald's omega coefficients (omega) (Revelle and Zinbarg 2009), was also tested. Factor score determinacy coefficients were also reported. Significance was set at < 0.05.

### 4.7 Ethical Considerations

The study was approved before the start of data collection by the Ethical Committee of Fondazione Policlinico Universitario Campus Bio‐Medico (PAR 45.22 OSS, June 22nd 2022). Confidentiality of participant identity and data protection were warranted in compliance with ethical principles of the Helsinki Declaration (World Medical Association 2013) and current regulations. Socio‐demographic and other data collected were pseudo‐anonymised, ensuring that individual participants could not be identified by the researchers. Potential participants were provided with detailed information about the study objectives and procedures at the beginning of the questionnaire. Participants' consent to study participation and data handling was required online before they could proceed to complete and return the questionnaire.

## 5 Results

### 5.1 Characteristics of the Sample

The 725 nurses who participated in the survey were mostly female (*n* = 552, 76.1%), on average 38.7 years (SD 11.33) old and with a mean work experience of 14.6 years (SD 11.03). They were mostly hospital‐based (*n* = 581, 80.1%), and prevalent clinical areas included surgical (*n* = 135, 18.6%), medical (*n* = 112, 15.4%), critical care/emergency (*n* = 73, 10.1%), palliative care (*n* = 93, 12.8%), oncology (*n* = 42, 5.8%), home care (*n* = 52, 7.2%) and hospital clinics (*n* = 33, 4.6%). Nurses were based in 13 different regions covering Central Italy (*n* = 390, 54.7%) Northern Italy (*n* = 187, 26.2%), Southern Italy, and the main Islands (*n* = 136, 19.1%). The most frequent religion reported by participants was Roman catholic (*n* = 587, 81%), although mostly not practising (*n* = 416, 57.4%). More details on participants' characteristics are provided in Table 1.

**TABLE 1:** Participants' characteristics (*n* = 725 nurses).

Variable	*N* (%)	Mean ± SD (range)
Gender		
Female	552 (76.1)	
Male	173 (23.9)	
Age (years)		38.7 ± 11.33 (22–67)
Education		
Diploma	168 (23.2)	
Bachelor	463 (63.9)	
Master	91 (12.6)	
Doctorate	3 (0.4)	
Work experience (years)		14.6 ± 11.03 (01–43)
Clinical setting		
Hospital	581 (80.1)	
Community	144 (19.9)	
Clinical area		
Surgical	135 (18.6)	
Medical	112 (15.4)	
Critical care/emergency	73 (10.1)	
Palliative care	93 (12.8)	
Oncology	42 (5.8)	
Hospital clinics	33 (4.6)	
Home care	52 (7.2)	
Other^a^	185 (25.5)	
Geographical area		
Central Italy	390 (54.7)	
Northern Italy	187 (26.2)	
Southern Italy and main Islands	136 (19.1)	
Religion		
Roman Catholic	587 (81)	
Orthodox	12 (1.7)	
Other Christian Church	11 (1.5)	
Muslim	2 (0.3)	
Jewish	1 (0.1)	
Other religion	20 (2.8)	
Atheist	65 (9)	
Agnostic	27 (3.7)	
Religious attendance		
Yes	309 (42.6)	
No	416 (57.4)	

Abbreviation: SD = standard deviation.

^a^ Other clinical areas include outpatient clinics and other services both hospital‐ and community‐based.

### 5.2 Structural Validity

The distribution of six items did not approach univariate normality, with skewness and kurtosis indices >|1| (see Table 2). To test structural validity, a CFA was performed on the whole sample (*n* = 725) by positing the 4‐factor model of the original instrument (Giske et al. 2023). In this model, the Intrapersonal Spirituality factor was measured by 7 items (#1‐#7), the Interpersonal Spirituality factor was measured by 5 items (#8‐#12), the Spiritual Care Assessment/Planning factor was measured by 8 items (#13‐#20), and the Spiritual Care Intervention/Evaluation factor was measured by 8 items (#21‐#28). The goodness‐of‐fit indices of the model revealed a misfit (see Table 3).

**TABLE 2:** EPICC Tool‐It item descriptive characteristics, factor loadings at CFA, scores, reliability and correlations.

Item		Mean	SD	Skew	Kurt	F1	F2	F3	F4	EPICC Tool‐It
1	I understand the concept of spirituality	4.001	0.606	−0.600	0.552	**0.585** *	0.176	0.067	−0.067	
2	I can explain the impact of spirituality on a person's health and well‐being across the lifespan for myself and others	3.716	0.697	−0.650	0.753	**0.806** *	−0.076	0.047	0.086	
3	I understand the impact of my own values and beliefs in providing spiritual care	3.977	0.649	0.796	0.998	**0.673** *	0.205	−0.017	0.038	
4	I reflect meaningfully upon my own values and beliefs and recognise that these may be different from other people's values and beliefs	4.295	0.603	−1.272	2.409	0.272	**0.568** *	−0.046	−0.194*	
5	I take care of my own well‐being	3.912	0.657	−0.755	1.092	0.104	**0.424** *	−0.046	0.158	
6	I am willing to explore my own personal, religious, and spiritual beliefs	4.022	0.648	−0.881	1.438	0.227	**0.407** *	0.052	0.013	
7	I am open and respectful to people's diverse expressions of spirituality	4.404	0.484	−1.156	1.942	−0.070	**0.783** *	0.018	−0.095	
8	I understand the ways that people express their spirituality	3.881	0.562	−0.450	0.532	0.043	**0.508** *	0.072	0.053	
9	I am aware of the different world/religious views and how these may impact upon people's responses to key life events	4.181	0.498	−0.553	0.240	0.037	**0.701** *	0.052	−0.157	
10	I recognise the uniqueness of people's spirituality	4.065	0.552	−0.569	0.339	0.034	**0.639** *	0.074	−0.120	
11	I interact with, and respond sensitively to people's spirituality	4.117	0.528	−0.765	1.235	−0.049	**0.667** *	0.151	0.085	
12	I am trustworthy, approachable, and respectful of people's expressions of spirituality and different world/religious views	4.199	0.498	−0.725	0.832	−0.093	**0.731** *	0.048	0.054	
13	I understand the concept of spiritual care	3.914	0.677	−0.718	0.840	**0.354** *	0.103	**0.491** *	0.063	
14	I am aware of different approaches to spiritual assessment	3.866	0.657	−0.654	0.656	0.064	0.135	**0.574** *	0.151	
15	I understand other professionals' roles in providing spiritual care	3.977	0.599	−0.691	0.838	0.034	0.288	**0.562**	0.011	
16	I can conduct and document a spiritual assessment to identify spiritual needs and resources	3.371	0.898	−0.361	−0.062	−0.035	−0.120	**0.394** *	**0.636** *	
17	I can collaborate with other professionals in the provision of spiritual care	3.702	0.783	0.720	0.702	0.075	0.032	0.350*	**0.560** *	
18	I can appropriately contain and deal with emotions	3.806	0.656	−0.707	0.854	0.031	**0.400**	−0.125	**0.397** *	
19	I am open, approachable, and non‐judgmental	4.211	0.500	−0.770	1.206	−0.148	**0.773** *	−0.148	0.127	
20	I am willing to deal with emotions	4.143	0.542	−0.834	1.418	0.188	**0.564** *	−0.246*	0.191	
21	I understand the concept of compassion and presence and its importance in spiritual care	4.210	0.447	−0.630	1.055	0.144	**0.589** *	0.151	−0.054	
22	I know how to respond appropriately to identified spiritual needs and resources	3.766	0.668	−0.395	0.117	0.062	0.281	0.008	**0.510** *	
23	I know how to evaluate whether spiritual needs have been met	3.559	0.754	−0.269	−0.090	0.021	0.170	0.047	**0.681** *	
24	I recognise my personal limitations in spiritual care giving and refer to others as appropriate	4.161	0.577	0.864	1.115	0.036	**0.429** *	0.090	0.138	
25	I evaluate and document personal, professional, and organisational aspects of spiritual care and reassess appropriately	3.560	0.900	−0.575	0.237	0.001	0.041	0.088	**0.705** *	
26	I show compassion and presence	4.230	0.450	−0.609	0.658	0.031	**0.593** *	0.078	0.000	
27	I am willing to collaborate with and refer to others (professional/non‐professional) in providing spiritual care	4.234	0.560	−1.102	2.116	0.167	**0.494** *	0.090	0.069	
28	I am welcoming and accepting and show empathy, openness, professional humility and trustworthiness in seeking additional spiritual support	4.302	0.454	−0.664	0.396	−0.033	**0.708** *	0.022	−0.060	
F1		3.90	0.683			1				
F2		4.14	0.481			0.671**	1			
F3		3.92	0.690			0.729**	0.707**	1		
F4		3.59	0.724			0.632**	0.615**	0.746**	1	
EPICC tool‐It		3.99	0.484			0.812**	0.787**	0.909**	0.800**	1
*α*						0.802	0.913	0.822	0.868	0.941
*ω*						0.804	0.916	0.823	0.869	0.859
FSD						0.915	0.958	0.934	0.936	0.924

*Note:* Item descriptive characteristics refer to the whole sample (*n* = 725 nurses), while factor loadings at CFA, scores, reliability and correlations refer to the subsample (*n* = 384 nurses). Primary loadings in bold.

Abbreviations: F1 = knowledge of spirituality, F2 = attitudes towards spirituality and spiritual care, F3 = knowledge of spiritual care, F4 = skills in spiritual care, FSD = factor score determinacy coefficient, Q1–Q28 = item number of EPICC Tool‐It, SD = standard deviation, skew = skewness; kurt = kurtosis, *α* = Cronbach's alpha coefficient, *ω* = omega Coefficient.

* *p* < 0.05.

** *p* < 0.01.

**TABLE 3:** Fit indices of exploratory and confirmatory factor analysis for all EPICC tool‐It models.

Model	*N*	*χ* ^2^	Df	*p*	CFI	TLI	RMSEA (90% CI)	SRMR
CFA original model	725	1.913.311	344	< 0.001	0.771	0.748	0.079 (0.076–0.083)	0.080
EFA^a^	384	630.428	272	< 0.0001	0.910	0.875	0.059 (0.053–0.065)	0.035
Initial CFA	346	737.150	340	< 0.0001	0.875	0.863	0.057 (0.052–0.063)	0.063
Final CFA	346	597.225	338	< 0.0001	0.918	0.908	0.047 (0.041–0.053)	0.060

*Note:* *N*: sample size.

Abbreviations: CFI, Comparative Fit Index; Df, degrees of freedom; RMSEA (90% CI): root mean square error of approximation with a 90% confidence interval; SRMR, standardised root mean square residual; TLI, Tucker‐Lewis Index; *χ* ^2^, chi‐squared.

^a^ Kaiser‐Meyer‐Olkin = 0.935; Bartlett's test of sphericity significant (*p* < 0.001).

Therefore, exploratory factor analysis (EFA) and subsequent CFA were planned by randomly splitting the sample into two subsamples (subsample 1, *n* = 384; subsample 2, *n* = 346). The Kaiser‐Meyer‐Olkin (KMO) measure of sampling adequacy was 0.935, and the Bartlett's test of sphericity was significant (*p* < 0.001). Therefore, the dataset was deemed suitable for factor analysis.

Parallel analysis suggested that a four‐factor solution was the most adequate. Accordingly, a four‐factor EFA was conducted; however, the goodness of fit indices were inadequate (see Table 3). The factors were labelled: Knowledge of spirituality (F1, measured by 3 items: #1, #2, #3), Attitudes towards spirituality and spiritual care (Factor 2, measured by 17 items: #4‐#12, #18‐#21, #24, #26‐#28), Knowledge of spiritual care (Factor 3, measured by 3 items: #13‐#15) and Skills in spiritual care (Factor 4, measured by 5 items: #16, #17, #22, #23, #25). All primary factor loadings were two times greater than the secondary loading, except for items #13, #16, #17, and #18, which cross‐loaded between factors (see Table 2). Table S1 (see Supporting Information 2) presents the reallocation of items from the original structure to the factor structure identified through EFA in this sample.

Therefore, this factorial pattern was tested with CFA on the second subsample, including 346 nurses.

**FIGURE 1:**
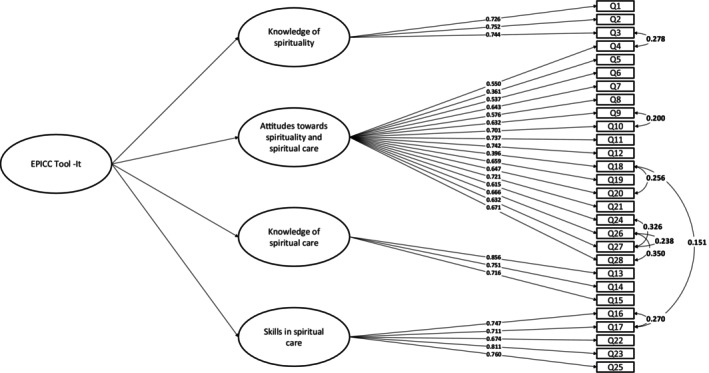
CFA figure of EPICC tool‐it.

The fit indices found a misfit (see Table 3). Following inspection of the modification indices (MI), we specified the covariance of adjacent items (#4 with #21, #18 with #20, #21 with #28, #23 with #25, #24 with #27, #26 with #28, #26 with #27, #27 with #28). Since the four factors were highly correlated (mean *r* = 0.679, range 0.615–0.746, *p* < 0.001) a second‐order model was tested. The fit indices for this model were adequate (see Table 3). All items showed loadings > 0.5 except for two items (#5 and #18 that loaded 0.361 and 0.414, respectively) and *p* values < 0.001 (see Figure 1).

### 5.3 Construct Validity

Participants reporting religious attendance compared with people not reporting it achieved higher scores in all factors (Knowledge of spirituality, Attitudes towards spirituality and spiritual care, Knowledge of spiritual care, and Skills in spiritual care) and in the overall EPICC Tool‐It (*p* < 0.001, *p* = 0.32, *p* = 0.001, p < 0.001 and *p* < 0.001, respectively). The scores of the factors Knowledge of spirituality and Knowledge of spiritual care were higher (both with p < 0.001) in the more experienced compared with less experienced nurses, and in older than in younger nurses (*p* = 0.016 and *p* = 0.003, respectively). The scores of Knowledge of spiritual care and Skills in spiritual care were slightly higher in nurses with postgraduate education compared with nurses with a bachelor (*p* = 0.036 and *p* = 0.031, respectively). Scores did not significantly differ between clinical settings (hospital or community), hospital clinical areas and different geographical areas. The hypotheses posed to test known group validity were then confirmed, supporting the construct validity of the EPICC Tool‐It.

### 5.4 Reliability

The values of alpha for the four factors (Knowledge of spirituality, Attitudes towards spirituality and spiritual care, Knowledge of spiritual care, Skills in spiritual care) and the overall scale (EPICC Tool‐It) were 0.802, 0.913, 0.822, 0.868 and 0.941, respectively. The omega coefficients were 0.804, 0.916, 0.823, 0.869 and 0.859 for Knowledge of spirituality, Attitudes towards spirituality and spiritual care, Knowledge of spiritual care, Skills in spiritual care and EPICC Tool‐It, respectively. Moreover, the factor score determinacy coefficients were 0.915, 0.958, 0.934, 0.936 and 0.924 for Knowledge of spirituality, Attitudes towards spirituality and spiritual care, Knowledge of spiritual care, Skills in spiritual care and EPICC Tool‐It, respectively (see Table 2). All these values showed excellent reliability.

## 6 Discussion

This study aimed to cross‐culturally adapt and psychometrically test the Italian version of the EPICC Self‐assessment tool of spiritual care competences for nurses (EPICC Tool‐It). Participants were a large sample of nurses across 13 regions (Northern, Central, South and main Islands) of Italy. Despite the sample's gender imbalance, skewed towards female nurses working in hospitals, their characteristics reflect the general distribution of nurses in many countries, including Italy (Heinen et al. 2013; Vitale et al. 2023). The original EPICC Tool (Giske et al. 2023) was cross‐culturally adapted to the Italian nursing context through a rigorous methodology. A cross‐validation with EFA and CFA was conducted, confirming the structural validity of EPICC Tool‐It as a 4‐factor model with satisfactory fit indices and high and significant loadings. The four factors found in the Italian version were consistent with the structure of the original EPICC Tool by underlying the skill, knowledge and attitude dimensions of the competence in spiritual care.

The covariances specified between item #4 (*I reflect meaningfully upon my own values and beliefs and recognise that these may be different from other people's values and beliefs*) and item #21 (*I understand the concept of compassion and presence and its importance in spiritual care*), as well as between item #21 and item #28 (*I am welcoming and accepting and show empathy, openness, professional humility*, *and trustworthiness in seeking additional spiritual support*), between item #26 (*I show compassion and presence*) and item #28, between item #26 and item #27 (*I am willing to collaborate with and refer to others ‐professional/non‐professional‐ in providing spiritual care*), and between item #27 and item #28, are justified by their adjacency within the same factor and the similarity of the concepts explored (e.g., compassion, presence, empathy, being welcoming and willing to collaborate, etc.). Similarly, the covariance between item #24 (*I recognise my personal limitations in spiritual care giving and refer to others as appropriate*) and #27 (*I am willing to collaborate with and refer to others ‐professional/non‐professional‐ in providing spiritual care*), the covariance between item #18 (*I can appropriately contain and deal with emotions*) and item #20 (*I am willing to deal with emotions*), and between item #23 (*I know how to evaluate whether spiritual needs have been met*) and item #25 (*I evaluate and document personal, professional, and organisational aspects of spiritual care, and reassess appropriately*), can be explained by semantic similarity and because they share the same concept (e.g., dealing with emotions, referring to others for spiritual care).

A hierarchical structure with a second‐order factor was also found. This is important because, besides considering individual factor scores, it will be possible to also calculate a single score depicting the level of self‐perceived spiritual care competence as a whole.

Construct validity was also established by analysis of known group validity. The hypotheses posed were confirmed as scores at EPICC Tool‐It factors varied in accordance with several participant characteristics (work experience, education and religiosity). The scores of all the EPICC Tool‐It factors were higher in those reporting a religious attendance, and the scores of factors regarding knowledge of spirituality and of spiritual care were significantly higher in nurses with longer work experience, in line with previous literature (Green et al. 2020; Hsieh et al. 2020; Kang et al. 2021; Ross et al. 2016). Moreover, the scores of Knowledge of spiritual care and Skills in spiritual care were significantly higher in nurses with postgraduate education compared with those with bachelor degrees, in accordance with Kang et al. (2021). The fact that more experienced and educated nurses scored higher in the dimensions regarding knowledge and skills in spiritual care is not surprising, as knowledge and skills are expected products of education and practical experience (Attard et al. 2014; Ross et al. 2018).

Interestingly, the dimension regarding nurses' attitudes towards spirituality did not differ with work experience and education. This can be explained by the essential feature of the attitude towards spirituality that depends on individual characteristics which are unrelated to experience or education (Baldacchino 2006; Ramezani et al. 2014). The score distribution of knowledge and skills in spiritual care behaved differently from that of attitudes towards spiritual care also in previous research validating the original EPICC Tool (Giske et al. 2023).

The distribution of EPICC Tool‐It scores was not statistically significant across healthcare settings (hospital and community) and hospital clinical areas, in line with previous literature (Mamier et al. 2019; Taylor et al. 2023; Van Leeuwen and Schep‐Akkerman 2015). Such results can perhaps be explained by the work organisation in Italy where nurses are often requested to move from one clinical area (in particular, medical/surgical areas) to another, and nurses' specialisation in a specific clinical area is an individual choice, not necessarily linked to receiving a preferred job contract in that area. Therefore, it is frequently possible to find nurses working in a specific clinical area without having gained specialised work experience able to foster spiritual care competences (Cone and Giske 2017).

Internal consistency of all the factors and of the overall scale was excellent. Therefore, EPICC Tool‐It is a multidimensional instrument showing good validity and excellent reliability. This justifies computing and using a score for each factor of EPICC Tool‐It and an overall score describing the level of nurses' self‐reported competence in providing spiritual care. Such a score can be standardised to 100 with the help of the following formula: EPICC Tool‐It = SUM (item1:item28; −28)/112 × 100. For instance, if the sum score of all the items (1–28) is 80, the calculation of the overall standardised score will be: EPICC Tool‐It = (80–28)/112 × 100 = 46.4.

### 6.1 Implications for Policy and Practice

This study provides nurse managers and clinical nurses with a valid and reliable tool for measuring nurses' self‐reported competencies in providing spiritual care. The EPICC Tool‐It should be used at regular intervals to identify the need for reinforcing education or training related to spiritual care, and to raise awareness among health professionals and managers about the importance of this often‐neglected aspect of patient care. Additionally, it could serve as a means to assess the impact of educational and awareness‐raising strategies aimed at developing the personal and professional competencies of the nursing workforce.

### 6.2 Strength and Limitations of the Study

The primary strengths of this study lie in the rigorous methodology used for the instrument's cultural adaptation and psychometric testing, as well as the large, nationwide sample. However, several limitations should be acknowledged. The EPICC Tool‐It is a self‐report instrument, meaning the competencies measured reflect nurses' perceptions and aspirations, which may not fully align with their actual practices. Although the study used convenience and snowball sampling, the sample characteristics (e.g., age, gender and work setting) were representative of the nursing population in Italy (Vitale et al. 2023). Lastly, the responsiveness and test–retest stability of EPICC Tool‐It were not investigated in this study.

### 6.3 Recommendations for Further Research

To further validate the utility of the EPICC Tool‐It, future research should focus on assessing its responsiveness and stability over time through longitudinal studies and test–retest reliability assessments. Additionally, replicating the study with nursing students would be useful to confirm the scale's validity and reliability in this population. By identifying specific areas of competence in spiritual care, the EPICC Tool‐It can inform curriculum development and continuing education programs, thereby ensuring a higher standard of holistic patient care.

## 7 Conclusion

The validation of the EPICC Tool‐It provides a robust measure for assessing the spiritual care competencies of Italian‐speaking nurses, addressing a critical gap in the field. Our findings support the promotion of equity, inclusion and multiculturalism in nursing competencies related to spiritual care, enhancing both the accessibility of the tool for Italian nurses and the representativeness of international research. The EPICC Tool‐It is a valuable instrument for both research and practice, enabling the assessment of self‐perceived spiritual care competencies and guiding targeted educational interventions.

## Author Contributions

**Michela Piredda:** conceptualisation, methodology, investigation, data curation, formal analysis, writing – original draft, writing – review and editing, visualisation. Methodology, formal analysis. **Alessio Lo Cascio:** validation, writing – original draft, writing – review and editing, visualisation. investigation, data curation, writing – original draft, visualisation. methodology, validation, writing – review and editing, visualisation. **Maddalena De Maria:** data curation, methodology, formal analysis, writing – original draft, writing – review and editing, visualisation. **Roberto Latina:** methodology, formal analysis, writing – original draft, writing – review and editing, visualisation. **Anna Marchetti:** writing – original draft, writing – review and editing, visualisation. **Anna De Benedictis:** methodology, formal analysis, writing – original draft, writing – review and editing, visualisation. **Giorgia Petrucci:** writing – original draft, writing – review and editing, visualisation. **Linda Ross:** writing – review and editing, visualisation. **Wilfred McSherry:** writing – review and editing, visualisation. **Maria Grazia De Marinis:** conceptualisation, supervision.

## Disclosure

Statistics: The authors state that the methods used in the data analyses are suitably applied to their data within their study design and context, and the statistical and psychometric findings have been implemented and interpreted correctly.

## Conflicts of Interest

The authors declare no conflicts of interest.

## Supporting information


Data S1.

Table S1.

## Data Availability

The study data (source dataset, input and output of analyses) are made publicly available in the Mendeley repository (https://data.mendeley.com/my‐data/) as: Piredda, Michela (2025), ‘Data and analyses cross‐validation EPICC Tool‐it,’ Mendeley Data, V1, doi: 10.17632/xj4hccbhm8.1
