# Supplementary material for: Cross‐Cultural Adaptation and Cross‐Validation of the Italian Version of the EPICC Spiritual Care Competency Self‐Assessment Tool for Clinical Nurses
Source: J Clin Nurs. 2025 Mar 18;34(11):4757–68. doi: 10.1111/jocn.17738 (PMC12489437; doi:10.1111/jocn.17738)
Supplement: Supplementary file 2 — Table S1. [file JOCN-34-4757-s002.docx]

| **Table 4 - Comparison between item loading on EPICC Tool-It factors and on EPICC Tool original factors** | | | | |
| --- | --- | --- | --- | --- |
| **Item n.** | | **Item content** | **IT factor** | **Original factor** |
| 1 | I understand the concept of spirituality | | Knowledge of spirituality | INTRApersonal Spirituality (Knowldege) |
| 2 | I can explain the impact of spirituality on a person’s health and well-being across the lifespan for myself and others | | Knowledge of spirituality | INTRApersonal Spirituality (Knowldege) |
| 3 | I understand the impact of my own values and beliefs in providing spiritual care | | Knowledge of spirituality | INTRApersonal Spirituality (Knowldege) |
| 4 | I reflect meaningfully upon my own values and beliefs and recognise that these may be different from other people’s values and beliefs | | Attitudes towards spirituality and spiritual care | INTRApersonal Spirituality (Skills) |
| 5 | I take care of my own well-being | | Attitudes towards spirituality and spiritual care | INTRApersonal Spirituality (Skills) |
| 6 | I am willing to explore my own personal, religious, and spiritual beliefs | | Attitudes towards spirituality and spiritual care | INTRApersonal Spirituality (Attitudes) |
| 7 | I am open and respectful to people’s diverse expressions of spirituality | | Attitudes towards spirituality and spiritual care | INTRApersonal Spirituality (Attitudes) |
| 8 | I understand the ways that people express their spirituality | | Attitudes towards spirituality and spiritual care | INTERpersonal Spirituality (Knowledge) |
| 9 | I am aware of the different world/religious views and how these may impact upon people’s responses to key life events | | Attitudes towards spirituality and spiritual care | INTERpersonal Spirituality (Knowledge) |
| 10 | I recognise the uniqueness of people’s spirituality | | Attitudes towards spirituality and spiritual care | INTERpersonal Spirituality (Skills) |
| 11 | I interact with, and respond sensitively to people’s spirituality | | Attitudes towards spirituality and spiritual care | INTERpersonal Spirituality (Skills) |
| 12 | I am trustworthy, approachable, and respectful of people’s expressions of spirituality and different world/religious views | | Attitudes towards spirituality and spiritual care | INTERpersonal Spirituality (Attitudes) |
| 13 | I understand the concept of spiritual care | | Knowledge of spiritual care | Spiritual Care Assessment and Planning (Knowledge) |
| 14 | I am aware of different approaches to spiritual assessment | | Knowledge of spiritual care | Spiritual Care Assessment and Planning (Knowledge) |
| 15 | I understand other professionals’ roles in providing spiritual care | | Knowledge of spiritual care | Spiritual Care Assessment and Planning (Knowledge) |
| 16 | I can conduct and document a spiritual assessment to identify spiritual needs and resources | | Skills in spiritual care | Spiritual Care Intervention and Evaluation (Skills) |
| 17 | I can collaborate with other professionals in the provision of spiritual care | | Skills in spiritual care | Spiritual Care Intervention and Evaluation (Skills) |
| 18 | I can appropriately contain and deal with emotions | | Attitudes towards spirituality and spiritual care | Spiritual Care Intervention and Evaluation (Skills) |
| 19 | I am open, approachable, and non-judgmental | | Attitudes towards spirituality and spiritual care | Spiritual Care Assessment and Planning (Attitudes) |
| 20 | I am willing to deal with emotions | | Attitudes towards spirituality and spiritual care | Spiritual Care Assessment and Planning (Attitudes) |
| 21 | I understand the concept of compassion and presence and its importance in spiritual care | | Attitudes towards spirituality and spiritual care | Spiritual Care Intervention and Evaluation (Knowledge) |
| 22 | I know how to respond appropriately to identified spiritual needs and resources | | Skills in spiritual care | Spiritual Care Intervention and Evaluation (Knowledge) |
| 23 | I know how to evaluate whether spiritual needs have been met | | Skills in spiritual care | Spiritual Care Intervention and Evaluation (Knowledge) |
| 24 | I recognise my personal limitations in spiritual care giving and refer to others as appropriate | | Attitudes towards spirituality and spiritual care | Spiritual Care Assessment and Planning (Skills) |
| 25 | I evaluate and document personal, professional, and organisational aspects of spiritual care, and reassess appropriately | | Skills in spiritual care | Spiritual Care Intervention and Evaluation (Skills) |
| 26 | I show compassion and presence | | Attitudes towards spirituality and spiritual care | Spiritual Care Intervention and Evaluation (Attitudes) |
| 27 | I am willing to collaborate with and refer to others (professional/non-professional) in providing spiritual care | | Attitudes towards spirituality and spiritual care | Spiritual Care Intervention and Evaluation (Attitudes) |
| 28 | I am welcoming and accepting and show empathy, openness, professional humility, and trustworthiness in seeking additional spiritual support | | Attitudes towards spirituality and spiritual care | Spiritual Care Intervention and Evaluation (Attitudes) |

*Legend: Item n.= item number; IT Factor= EPICC Tool-It factor.*
